# A Randomized Controlled Trial on the Efficacy of 20% Human Albumin in Reducing Pleural Effusion After Cardiopulmonary Bypass

**DOI:** 10.3390/jcm13247693

**Published:** 2024-12-17

**Authors:** Kaspars Setlers, Klaudija Aispure, Maksims Zolovs, Ligita Zvaigzne, Olegs Sabelnikovs, Peteris Stradins, Eva Strike

**Affiliations:** 1 Department of Cardiovascular Anesthesia and Intensive Care, Pauls Stradins Clinical University Hospital, LV-1002 Riga, Latvia; 2 Department of Anesthesiology, Riga Stradins University, LV-1007 Riga, Latvia; 3 Faculty of Medicine, Riga Stradins University, LV-1007 Riga, Latvia; 4 Statistics Unit, Riga Stradins University, LV-1007 Riga, Latvia; 5 Institute of Life Sciences and Technology, Daugavpils University, LV-5401 Daugavpils, Latvia; 6 Institute of Radiology, Pauls Stradins Clinical University Hospital, LV-1002 Riga, Latvia; 7 Department of Intensive Care, Pauls Stradins Clinical University Hospital, LV-1002 Riga, Latvia; 8 Department of Cardiac Surgery, Pauls Stradins Clinical University Hospital, LV-1002 Riga, Latvia; 9 Department of Surgery, Riga Stradins University, LV-1007 Riga, Latvia

**Keywords:** cardiopulmonary bypass, cardiac surgery, pleural effusion, hypoalbuminemia

## Abstract

**Background/Objectives**: Cardiopulmonary bypass can lead to hemodilution, causing a fluid shift to the interstitial space. Albumin helps counteract the intravascular fluid movement to the extravascular space and reduces the risk of complications associated with fluid imbalance. Our main objective was to evaluate the effectiveness of albumin addition in the cardiopulmonary bypass priming solution compared to standard priming, focusing on its role in reducing pleural effusion development. **Methods**: This was a single-center randomized controlled trial conducted at a tertiary care hospital specializing in cardiology and cardiac surgery. It involved 70 individuals scheduled for elective open-heart surgery. All cases were randomly assigned into two groups of 35 patients. The study group replaced 100 mL of standard CPB priming solution with 100 mL of 20% human albumin. We measured serum albumin levels before and after the surgery, 6 and 12 h after, and calculated colloid oncotic pressure. Thorax CT scans were performed on the first postoperative day to measure and calculate pleural effusion volume. **Results**: Albumin addition to cardiopulmonary bypass priming solution led to a significant reduction in pleural effusion development after CPB. An albumin level <35 g/L after the surgery showed a significant increase in pleural effusion development, and 100 mL of 20% albumin was sufficient to maintain serum albumin levels > 35 g/L. **Conclusions**: Our study suggests a link between postoperative hypoalbuminemia and the early development of pleural effusion after CPB, as well as the possible benefits of adding 100 mL of 20% albumin compared to standard crystalloid CPB priming to minimize postoperative pleural effusion development.

## 1. Introduction

Cardiopulmonary bypass (CPB) is a surgical technique that temporarily replaces heart and lung functions during specific cardiac and thoracic procedures, such as coronary artery bypass grafting (CABG) or heart valve replacement. Cardiopulmonary bypass can induce significant hemodynamic changes, affecting the patient during the surgery and in the postoperative period. The ischemic period followed by reperfusion associated with CPB triggers a systemic inflammatory response, leading to heightened vascular permeability and increased arteriolar resistance, which can result in pulmonary hypertension. Increased permeability can also shift fluid to the interstitial space, resulting in postoperative pulmonary complications (PPCs) like pulmonary edema and pleural effusion [1,2,3,4].

Another factor contributing to PPCs is the onset of cardiopulmonary bypass-caused hemodilution. Due to the initiation of CPB, the patient’s plasma is rapidly diluted by the prime volume of the bypass machine, and colloid oncotic pressure may decrease considerably, depending on the composition of the priming solution [2]. The choice of prime fluid for CPB remains a subject of debate. Usually, two main categories of prime solutions are employed: crystalloids and colloids. During CPB, a primary objective is to minimize fluid shift to the interstitial space to limit extracardiac complications as much as possible [1,2]. Human serum albumin, a colloid from human blood, serves various essential bodily roles. It can reduce systemic inflammatory responses, protect the endothelial glycocalyx, and prevent endothelial dysfunction. It contributes significantly to maintaining plasma oncotic pressure as a major plasma protein [5,6]. Hemodilution can affect albumin concentration, leading to an imbalance between hydrostatic and oncotic pressures across pulmonary capillaries. This imbalance promotes fluid translocation from the intravascular compartment to the interstitial and pleural spaces, culminating in the possible development of pleural effusion [5,6,7]. Hypoalbuminemia is a frequent complication after cardiac surgery. Up to 60% of all patients after cardiac surgery suffer from moderate to severe hypoalbuminemia in the first 24 h after surgery, and it is a summation of different factors depending on patient characteristics and perioperative factors [8]. Adding albumin to the priming solution can help reduce hemodilution and consequent extracardiac complications by maintaining colloid oncotic pressure. Albumin helps counteract the intravascular fluid shift to the extravascular space and reduces the risk of complications associated with fluid imbalance [7,9].

Postoperative pulmonary complications following CPB can significantly impact postoperative outcomes [2,3,10]. Patients who develop PPCs have prolonged mechanical ventilation, extended hospitalization, longer ICU stays, and elevated postoperative mortality [3,10,11,12]. One of the most common PPCs following CPB is pleural effusion [12].

Our primary objective was to evaluate the effectiveness of adding 100 mL of 20% human albumin to the CPB priming solution compared to standard priming, with a specific focus on its potential role in reducing the occurrence of pleural effusion. Our secondary objective was to assess the impact of albumin infusion on respiratory function. This study hypothesized that adding 100 mL of 20% human albumin to the CPB priming solution could reduce early postoperative pleural effusion development.

## 2. Materials and Methods

### 2.1. Design

This study was a prospective single-center randomized controlled trial performed at Pauls Stradins Clinical University Hospital (Riga, Latvia), a tertiary care hospital specializing in cardiology and cardiac surgery, from September 2022 to December 2023. The study was registered on Clinicaltrials.gov (Identifier: NCT06681415) and approved by the State Agency of Medicines of the Republic of Latvia on 20 September 2021 for a drug usage observation study. The study was approved by the medical ethics committee of Pauls Stradins Clinical University Hospital (Chairperson Prof. P. Stradins) under reference number 260821-11L, dated 26 August 2021. All participants provided written informed consent before enrolment.

### 2.2. Patients

The study targeted 70 individuals scheduled for elective open-heart surgeries, encompassing procedures such as ascending aorta surgery, coronary artery bypass grafting, and heart valve replacement or repair, either independently or in combination, without the use of hypothermic circulatory arrest. We did not include patients with a history of reduced left ventricular ejection fraction (EF < 50%), chronic kidney disease, chronic lung disease, pre-existing anemia, or pathological chest X-ray findings before surgery. Randomization was conducted using a web-based, computer-generated random sequence. Patients were allocated into two groups—those receiving an additional 100 mL of 20% human albumin and those receiving standard CPB priming solution—in a 1:1 ratio.

### 2.3. Interventions

We used a CPB priming volume of 1050 mL, comprising an isochloremic solution of Deltajonin^®^ (Pharma Gmbh, Trittau, Germany) and 250 mL of 15% Mannitol. The study group replaced 100 mL of Deltajonin^®^ solution with 100 mL of 20% human albumin. We used cold crystalloid cardioplegia in all cases. Patients were managed with mechanical ventilation per local protocols, utilizing an FiO_2_ of 0.6, a tidal volume of 6 mL/kg, and a positive end-expiratory pressure (PEEP) set at 5 mmHg. After extubation, oxygen was administered via a non-rebreathing face mask at a flow rate of 10 L/min. Blood gas analysis (pO_2_, pCO_2_, FiO_2_/O_2_ ratio, A–a gradients, Lactate) followed the local protocol. Serum albumin levels were measured preoperatively, 6 and 12 h post-operation. Normal albumin was defined as ≥35 g/L, mild as 30–35 g/L, moderate as 25–30 g/L, and severe hypoalbuminemia was defined as <25 g/L. Colloid oncotic pressure (COP) was calculated using the formula by J C Hoefs: COP = A (1.058 G + 0.163 A + 3.11) [13,14].

Thorax CT scans were performed on all patients on the first postoperative day, with subsequent image analysis conducted by a single radiologist. The radiologist measured pleural effusion size (cm), and we calculated effusion volume (mL) using the formula, Volume = 0.365 × b^3^ − 4.529 × b^2^ + 159.723 × b − 88.377, by Hazlinger et al. [15], where b is the maximum effusion depth measured in the axial (transverse) imaging plane. If patients were hemodynamically unstable with ST-segment abnormalities, we reused their transportation to a CT scan.

### 2.4. Outcomes

The primary outcomes were the effect of albumin addition on serum albumin level in the early postoperative period and the development of pleural effusion.

The secondary outcome measure was the effect of albumin addition on respiratory function, measured by blood gas analysis, in the early postoperative period.

### 2.5. Statistical Data Analysis

The assumption of data distribution was assessed by the Shapiro–Wilk test and inspection of the normal Q-Q plots. The assumption of sphericity was tested by Mauchly’s test and equality of variance by Levene’s test. The assumption of equality of covariance matrices was assessed by Box’s M test. The study employed a two-way mixed ANOVA test to investigate the potential interaction effect between albumin infusion and time (before surgery, 6 h after surgery, and 12 h after surgery), specifically examining their combined impact on blood albumin levels and COP. The post hoc analysis (main effects) was conducted by applying the Tukey adjustment.

A one-way MANCOVA was conducted to determine whether there were any statistically significant differences between the adjusted means of the combined right and left sides of pleural effusion (cm and mL) between albumin infusion, after controlling for the effect of the covariates (albumin and COP before surgery). All statistical analyses were performed by using the core package of Jamovi statistical software Ver. 2.5 (https://www.jamovi.org, accessed on 15 December 2024). An alpha level of 0.05 was used for all the statistical analyses. Despite the within-subjects nesting of the data, the between-factor nature of the design precluded the necessity for *p*-value correction. The power of analysis was 0.998, as assessed by the G*Power program 3.1.9.7.

## 3. Results

**Figure 1:**
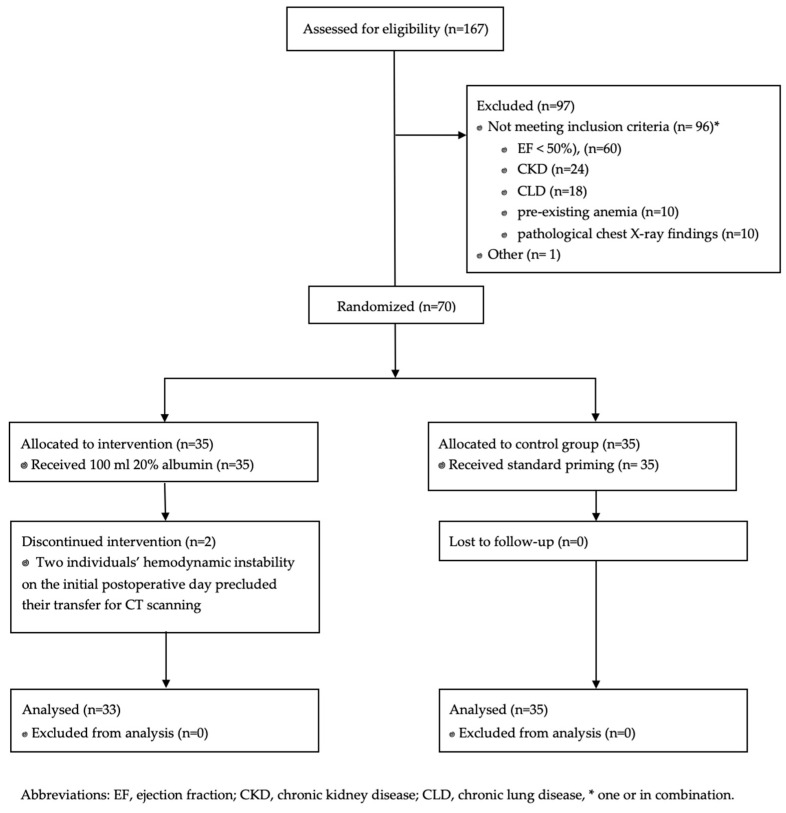
Study flow chart.

A total of 70 patients underwent analysis. Two individuals’ hemodynamic conditions on the initial postoperative day precluded their transfer for CT scanning. Both patients had received albumin infusion (Figure 1). The preoperative and intraoperative characteristics are shown in Table 1.

### 3.1. Primary Outcomes

There was no interaction between albumin addition and time on the level of albumin in the blood, F(2, 136) = 3.04, *p* = 0.051, indicating that the effect of infusion on the level of albumin in the blood is the same for 6 and 12 h after surgery.

The analysis of the main effects of the two-way mixed ANOVA showed that the level of albumin in the blood does not differ depending on albumin addition to the priming solution, F(1, 68) = 2.55, *p* = 0.115, whereas it decreases over time, F(2, 136) = 304.76, *p* < 0.001, η_G_^2^ = 0.62 (high effect size). Tukey post hoc analysis revealed that the level of albumin is significantly higher before surgery (mean = 45.0, 95% CI 44.2–45.9) than 6 h after surgery (mean = 36.4, 95% CI 35.6–37.2) and 12 h after surgery (mean = 36.2, 95% CI 35.5–36.9), *p* < 0.001.

**Figure 2:**
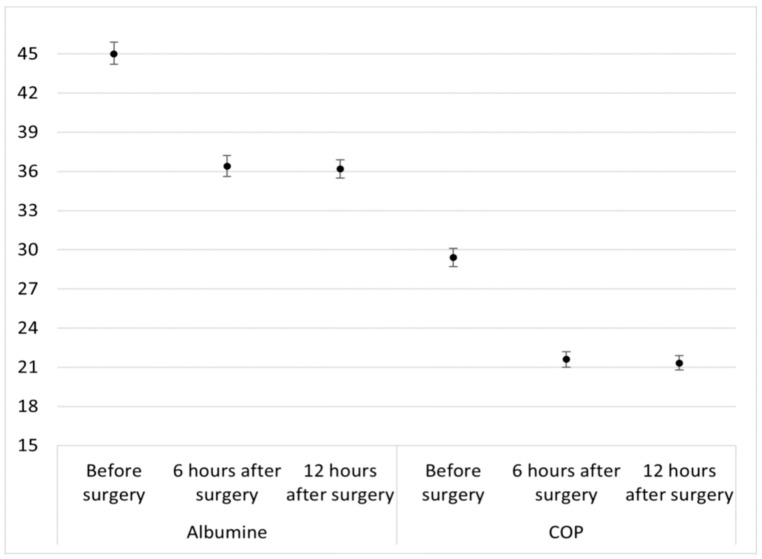
Time course of serum albumin level (g/L) and colloid oncotic pressure (mmHg) before and after cardiopulmonary bypass. COP, colloid oncotic pressure.

Similar results were obtained for COP. There was no interaction between albumin infusion and time on the COP, F(2, 136) = 1.80, *p* = 0.169, indicating that the effect of infusion on the level of COP is the same 6 and 12 h after surgery. The analysis of the main effects of the two-way mixed ANOVA showed that the COP does not differ depending on albumin infusion, F(1, 68) = 1.13, *p* = 0.291, whereas it differs depending on time, F(2, 136) = 320.03, *p* < 0.001, η_G_^2^ = 0.68 (high effect size). The level of COP is significantly higher before surgery (mean = 29.4, 95% CI 28.7–30.1) than 6 (mean = 21.6, 95% CI 21.0–22.2) and 12 h after surgery (mean = 21.3, 95% CI 20.8–21.9), *p* < 0.001 (Figure 2).

**Figure 3:**
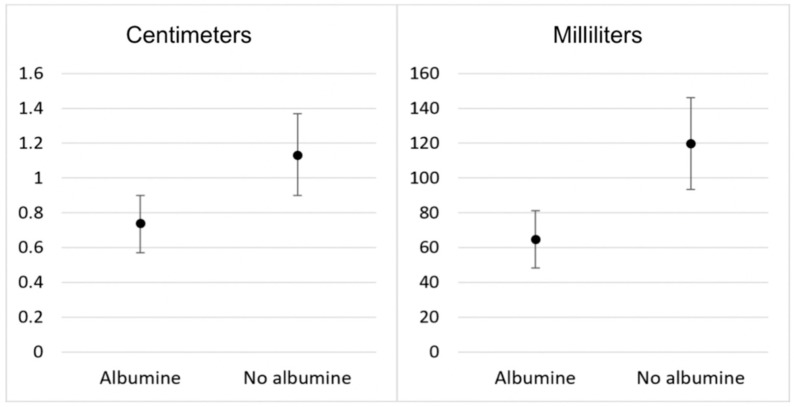
Pleural effusion (centimeters) in thoracic CT images and calculated in milliliters [volume 0.365 × b^3^ − 4.529 × b^2^ + 159.723 × b − 88.377, by Hazlinger et al.]. b—effusion depth (cm).

The covariates (albumin and COP before surgery) were not significantly related to pleural effusion, *p* > 0.05. The one-way MANCOVA was significant (Λ = 0.197, F(4, 61) = 3.76, *p* = 0.008), indicating significant differences in pleural effusion between albumin infusion after controlling for the effect of the covariates (albumin and COP before surgery) (Figure 3).

An albumin level of <35 g/L showed a significant impact on pleural effusion development (Table 2). No patient reached an albumin level of <30 g/L. The administered albumin volume (100 mL) was sufficient to maintain a normal albumin level (>35 g/L) after surgery (*p* < 0.05), as assessed by a one-sample Wilcoxon signed-rank test.

### 3.2. Secondary Outcomes

There was no interaction between albumin infusion and the blood gas analysis findings (pO_2_, pCO_2_, FiO_2_/O_2_ ratio, A–a gradients, Lactate), *p* > 0.05, indicating that the effect of infusion on blood gases is the same 6 and 12 h after surgery. The analysis of the main effects of the two-way mixed ANOVA showed that blood gases do not differ depending on albumin infusion, *p* > 0.05.

## 4. Discussion

The etiopathogenesis of pleural effusion following CPB involves several interrelated mechanisms, including the systemic inflammatory response, alterations in oncotic pressure, surgical factors, fluid overload, and the disruption of lymphatic drainage. Serum albumin levels may modulate the inflammatory response and oncotic pressure changes. In these cases, albumin supplementation could potentially improve outcomes by addressing these factors, particularly by decreasing excessive fluid accumulation and stabilizing vascular permeability. Our study aimed to compare the possible benefits of adding a small amount of relatively available and cheap albumin solution to CPB priming to maintain plasma colloid oncotic pressure and reduce fluid’s extravasation and possible accumulation in the lungs. Our question was whether, by adding a minimal amount of extra albumin to the CPB priming solution, we would still secure the patient from possible hypoalbuminemia and low COP-caused pleural effusion.

We compared two randomly selected groups of 35 patients who either received an additional 100 mL of 20% human albumin to their CPB priming or received standard crystalloid priming solution. Our findings show that hypoalbuminemia had a significant impact on pleural effusion development. The albumin volume (100 mL) used in our study was sufficient to maintain a normal albumin level (>35 g/L) after surgery. After controlling for the effect of the covariates (albumin and COP before surgery), we found significantly smaller pleural effusion volume in the albumin group.

Our study observed that the serum albumin level in the blood did not differ depending on albumin addition to the priming solution when assessed in absolute terms. Still, a decrease in albumin levels before surgery was observed compared to 6 and 12 h after surgery. A decline in serum albumin concentration may result from various factors, including dilution due to fluid resuscitation, enhanced catabolic activity, reduced hepatic synthesis, blood loss, CPB, and redistribution caused by inflammation-induced alterations in vascular permeability [1,2,16]. Berbel-Franco et al. conducted a prospective observational study with 2818 patients to evaluate the influence of serum albumin level on the outcome of cardiac surgery and found that the presence of postoperative hypoalbuminemia after cardiac surgery is frequent and the degree of hypoalbuminemia may be associated with worse outcomes, even in the long term [8].

The use of albumin-containing solutions for priming has been debated due to concerns regarding its possible benefits to significant major adverse events (MAE) and postoperative mortality. A systematic review and meta-analysis involving 42 randomized controlled trials comparing albumin infusion with synthetic colloids and crystalloids found no association between intravenous albumin use and differences in morbidity and mortality [17]. Also, a large randomized controlled trial demonstrated that using a 4% albumin solution for priming did not significantly reduce the risk of MAEs over 90 days compared to Ringer acetate [18]. One of the most common secondary adverse events observed in this study was pleural effusion, leading to ICU or hospital readmission, with rates of occurrence being (7 [1.0%] in the albumin group and 9 [1.3%]) in the Ringer group. The total incidence of pleural effusion following CPB varies from 10% to 40% [19]. Our recently published findings demonstrated a strong link between the development of left-sided pleural effusion and postoperative hypoalbuminemia (≤35 g/L) [20]. These results are consistent with a recent study by Liu et al., which identified serum albumin levels below 40 g/L as independent risk factors for the occurrence of postoperative pulmonary complications [11].

In our study, the analysis of albumin levels in absolute values and COP after CPB showed no difference based on albumin addition to the priming solution. Also, the covariates beforehand (albumin and COP before surgery) were not significantly related to pleural effusion, although an SAL of < 35 g/L significantly impacted the development of pleural effusion following CPB. After controlling for the effect of the covariates (albumin and COP before surgery), we found a significant difference in the reduction in pleural effusion development after albumin infusion. In our hospital setting, a colloid osmometer was inaccessible for measuring COP. Therefore, we resorted to calculating COP using the formula proposed by J C Hoefs, (COP = A (1.058 G + 0.163 A + 3.11) [14]. We chose this formula for COP calculation based on a study by Khazaei et al., in which the formula showed the most similar results to the osmometer [13]. We found that the administered volume of albumin (100 mL, 20%) was effective in maintaining postoperative serum albumin levels within the normal range (>35 g/L), with statistical significance (*p* < 0.05). Other studies typically utilize 4 or 5% albumin solutions for CPB priming, aiming for a colloid osmotic composition resembling physiological levels. Klanderman et al. conducted a study that measured and compared the COP of various blood products and colloids. Their findings indicated that 4% of albumin had a COP of 12.8 mmHg, while 20% had a COP of 73.5 mmHg [21]. However, it is essential to consider that the 20% albumin solution, when diluted to the standard CPB priming solution volume used in our study (1050 mL), results in an approximately 2% albumin solution. Thus, although our study used a lower albumin concentration than typical CPB priming solutions, the volume of 20% albumin administered was still sufficient to keep postoperative serum albumin levels within the normal range. This underscores the need to consider the concentration and volume of albumin administered in clinical settings. Verheij et al. conducted a study to investigate the impact of colloid infusion on COP and its relationship with fluid shifting into the lungs. Their findings suggested that plasma COP effectively kept colloid fluids, at least temporarily, within the plasma volume compartment, with less extravasation into the extrapulmonary extravascular space than saline [22]. Despite that, the increased extravascular accumulation in saline compared with colloid fluids did not include the lungs, possibly showing the predominant effect of hydrostatic pressure rather than of COP [22,23]. In contrast to other studies, a comparative analysis assessed the effects of priming CPB circuits with human albumin versus Ringer lactate on colloid osmotic pressure and extravascular lung water. The study found a significant postoperative rise in lung water among patients who did not receive colloid-based priming. However, as in our research, there was no apparent effect on respiratory function, leaving the clinical significance of this finding to be clarified [2]. Another study compared 100 off-pump coronary artery bypass (OPCAB) procedures with 97 conventional coronary artery bypass grafting (CABG)/CPB procedures. This investigation found no significant differences in postoperative chest radiographs between the two groups [24]. Consequently, this prompts us to question the role of cardioplegia-caused hemodilution in the early development of pleural effusion following CPB.

To our knowledge, our study represents one of the first studies analysing CT scan data to compare early pleural effusion after cardiac surgery. Pleural effusion development within the first 30 postoperative days following cardiac surgery is classified as early and believed to be directly related to the surgery [25]. Our study specifically aimed to assess the appearance of pleural effusions within the first 24 h post-cardiac surgery. We analysed procedures such as ascending aorta surgery, coronary artery bypass grafting, and heart valve replacement or repair, independently or in combination. CABG surgeries were not separated in our study. Post-CABG pleural effusion complicates about one-half to two-thirds [26] of cases, but the role of the surgery type in pleural effusion development has been controversial. Labidi et al. compared 2892 cardiac surgery patients and showed that valve replacement was more strongly associated with postoperative pleural effusions than CABG [27]; however, apparently, most studies favour the theory that internal mammary artery harvesting is associated with a higher prevalence of pleural effusion [27,28,29]. In a study, chest radiographs obtained 28 days after cardiac surgery showed a significantly higher rate of effusions among patients who had undergone either CABG surgery or combined CABG and valve surgery than those who had undergone valve replacement alone. However, the prevalence of large effusions was 10.9% in patients who received an internal mammary artery versus 4.5% in the patients who received only a saphenous vein graft, and this difference did not reach statistical significance [30].

While the length of stay in the ICU and hospital was not specifically assessed in our study, existing research indicates that pleural effusion is associated with prolonged ICU and hospital stays and increased ventilation time [8,29]. This suggests that pleural effusion may contribute to postoperative complications and necessitate extended medical care, potentially impacting the overall recovery process following cardiac surgery.

In our study, there was no interaction between albumin infusion and the blood gas analysis findings (pO_2_, pCO_2_, FiO_2_/O_2_ ratio, A–a gradients, Lactate). The lack of significant differences in respiratory function may be associated with the fact that fluid volume with a significant clinical impact may be individual, and possible respiratory insufficiency could be disguised by excessive oxygen supply delivered to all patients after extubation.

The study group that did not receive albumin showed significantly higher BMI before surgery, 28.6 (SD = 5) vs. 31.5 (SD = 3). This inequality may have impacted our results. For individuals with a higher BMI, there is often an excessive release of proinflammatory cytokines into the circulation, indicating that inflammatory processes are more severe [31]. Also, higher BMI often increases baseline fluid retention, particularly interstitial fluid, leading to a greater susceptibility to pleural effusion due to fluid overload [32]. Increased BMI can also impair lymphatic drainage and may exacerbate pleural effusion development postoperatively [33]. However, no pleural effusion or respiratory insufficiency were detected in any patient analysed before surgery.

Low albumin and COP levels may induce pleural effusion development, which could potentially become clinically significant, necessary to drain, and affect the postoperative period after the first day. Our study group patients had no major comorbidities that could affect fluid accumulation and respiration function (decreased EF, CKD, CLD). Therefore, more studies should be conducted using patients with more comorbidities.

### Limitations

This study has several limitations. First, as a single-center trial with a small sample size, the generalizability of the findings is restricted and may not be extended to all cardiac surgery patients. Second, using calculated colloid oncotic pressure introduces a potential source of error, as concerns have been raised in the literature regarding its accuracy. Third, the data analysis does not differentiate between types of CABG procedures, limiting our ability to attribute pleural effusion solely to surgical trauma associated with internal mammary artery graft harvesting. Fourth, we cannot rule out fluid drainage from the chest cavity during the early postoperative period for the few patients who had postoperative pleural drains. These limitations highlight the need for further research to delineate better the specific factors contributing to pleural effusion development after cardiac surgery.

## 5. Conclusions

Pleural effusion continues to pose a frequent challenge following cardiac surgery. Considering its overlapping etiology, it remains challenging to address all the factors involved in pleural effusion development and their importance. Our results show that 100 mL of 20% albumin added to a cardiopulmonary bypass priming solution was beneficial in maintaining serum albumin levels within the normal range in the early postoperative period. Our study suggests a link between albumin addition to CPB priming solution and the reduction in early pleural effusion development.

## Figures and Tables

**Table 1:** Patient preoperative and operative characteristics.

	Albumin (+) (35 Patients; 50%)	Albumin (−) (35 Patients; 50%)	*p*-Value
Age (mean; SD)	66.7 (12)	69.3 (8)	0.119
Men (No. of patients; No. %)	23 (66)	27 (77)	0.184
Women (No. of patients; No. %)	12 (34)	8 (23)	0.281
BMI (mean; SD)	28.6 (5)	31.5 (3)	0.027
Type of surgery (No. %)			
Coronary artery bypass graft (only)	15 (43)	20 (57)	0.480
CABG + valve replacement surgery	2 (6)	3 (9)	0.648
1 valve replacement surgery (only)	20 (57)	16 (46)	0.346
2 valve replacement surgery (only)	0 (0)	1 (3)	0.321
Preoperative, (mean; SD)			
Hb (g/L)	138.2 (16)	138.8 (14)	0.839
Hct (%)	41 (5)	40 (4)	0.953
Albumin (g/L)	45.2 (3)	45.6 (3)	0.895
Total protein (g/L)	70.1 (4)	71.2 (6)	0.887
Creatinine (µmol/L)	83.3 (18)	93.8 (17)	0.030
Intraoperative (mean, SD)			
Time of cardiopulmonary bypass (min)	93 (30)	93 (31)	0.731
Volume of cardioplegia (mL)	1914 (565)	1758 (492)	0.314
Diuresis (mL)	1089 (482)	1334 (761)	0.120
Postoperative fluid balance (mL)	+870 (546)	+782 (541)	0.724
Postoperative Hct (%)	27 (3)	29 (3)	0.041

**Table 2:** Relation of Pleural Effusion and Serum Albumin Levels After Surgery.

Pleural Effusion	Albumin Level 6 h After Surgery		*p*	Albumin Level 12 h After Surgery		*p*
	≥35 g/L Median (Q1–Q3)	30–34 g/L Median (Q1–Q3)		≥35 g/L Median (Q1–Q3)	30–34 g/L Median (Q1–Q3)	
Right side (cm)	0.8 (0–1.0)	1.0 (0.9–2.0)	0.001	0.8 (0–1.0)	1.5 (1.0–2.0)	<0.001
Right side (mL)	36.7 (24.2–67.0)	67.0 (51.9–215.0)	0.002	36.7 (23.4–67.0)	142.0 (67.0–215.0)	<0.001
Left side (cm)	0.8 (0–1.0)	1.0 (0.9–1.0)	0.002	0.8 (0–1.0)	2.0 (1.0–2.0)	<0.001
Left side (mL)	36.7 (24.4–67.0)	67.0 (51.9–215.0)	0.004	36.7 (23.4–67.0)	215.0 (67.0–215.0)	<0.001

Relation of pleural effusion size (cm) and volume (mL) on thorax CT scan and its connection to serum albumin level in patient groups with normal serum albumin level and mild hypoalbuminemia, 6 and 12 h after surgery.

## Data Availability

The raw data supporting the conclusions of this article will be made available by the authors on request.
